# Supplementary material for: Flexible, sticky, and biodegradable wireless device for drug delivery to brain tumors
Source: Nat Commun. 2019 Nov 15;10:5205. doi: 10.1038/s41467-019-13198-y (PMC6858362; doi:10.1038/s41467-019-13198-y)
Supplement: Supplementary file 1 — Supplementary Information [file 41467_2019_13198_MOESM1_ESM.docx]

Supplementary Information

**Flexible, Sticky, and Biodegradable Wireless Device for Drug Delivery to Brain Tumors**

Jongha Lee *et al.*

**Supplementary Methods**

- 1. **Theoretical analysis of the wireless thermal actuation**

Electromagnetic simulations were performed using High Frequency Electromagnetic Field Simulation (HFSS) software to reveal the eddy current distribution following the external wireless mild-thermic actuation. The magnetic field generated from the transmission coil can be calculated as follows. The magnetic field intensity (*B_1_*) generated from the coil is expressed as,

$$B_{1}=\frac{\mu_{0}I a^{2}}{\left( a^{2}+h^{2} \right)^{1.5}} (1)$$

where *h* is the distance from the center of the coil, *a* is the radius of the coil, *I* is the current of the coil, and *μ_0_* is the permeability of vacuum. If the coil has several turns, the total sum of the magnetic field (*B*) can be calculated as,

$$B=\sum_{i=1}^{N} \frac{\mu_{0}I a^{2}}{\left( a^{2}+h_{i}^{2} \right)^{1.5}} (2)$$

where *N* is the turn number of the coil. For simplification, the distances from each turn of the coil to the heater, *h*_i_, can be considered to be same as *h.* Then the equation becomes as follows,

$$B=N\frac{\mu_{0}I a^{2}}{\left( a^{2}+h^{2} \right)^{1.5}} (3)$$

Using Supplementary Equation 3, the relation between the magnetic field and the temperature increase can be plotted as shown in Supplementary Fig. 13c. We assumed the magnetic field to be constant over the small area of the heater with the diameter of 12 mm at the distance of 3 cm from the coil (Quasi-static assumption). Internal layers between the transmission coil and the heater are modeled as a nonmagnetic material. Then the eddy current is calculated as,

$$J_{\phi}\left( \rho\right)=\frac{\sigma\omega B\left( \rho\right)\rho}{2} (4)$$

where *J* is the eddy current, *ρ* is the distance from the center of the heater, *σ* is the electrical conductivity of the Magnesium, *ω* is the frequency of the coil. Although the eddy current of the BEP with holes cannot be solved analytically, a simple assumption, the round heater model without holes, can be introduced for identifying the relationship between variables. Then, the Joule heating amount is as follows,

$$W=\int\frac{J_{\phi}^{2}}{2\sigma}dv=\frac{\pi\sigma t \omega^{2}B^{2}D^{4}}{256}=\frac{N^{2}\omega^{2}\mu_{0}^{2}I^{2} a^{4}D^{4}}{\left( a^{2}+h^{2} \right)^{3}}\frac{\pi\sigma t}{256} (5)$$

where *W* is the amount of the heat generation that is determined by the coil-to-heater distance (*h*), current of the transmission coil (*I*), number of the coil turn (*N*), coil radius (*a*), RF frequency ($\omega$), heater thickness (t), and heater diameter (*D*). According to the Supplementary Equation 5, the heat generation is proportional to the square of the coil current and the square of the RF frequency. Since *N, a,* and $\omega$ are fixed by the instrument (magnetic field generator), the key design parameters are the thickness (***t***) and diameter (***D***) of the wireless heater. The other parameters, coil-to-heater distance (h) and current in transmission coil (I), can also be optimized during the experiment.

For the analysis of the round heater with the hole array, the HFSS simulation is used. The heaters of three different diameters show similar amount of heat generation under the three different frequencies, which shows the potential for further minimization of the wireless heater (Supplementary Fig. 13e and f).

- 1. **Modeling of the three-dimensional (3D) thermal profile and DOX diffusion.**

***Finite element modeling of 3D thermal profiles at various heater temperatures.*** Finite element simulations were performed using COMSOL5.2 to reveal the temperature distribution following wireless mild-thermic actuation of the heater to accelerate drug delivery.

The brain tissue was simplified to be only represented by white matter, which consists of 75% (*w/w*) water, 9% (*w/w*) protein, and 16% (*w/w*) fat. After calculating the effective thermophysical properties of this tissue, we simply modeled it as a single layer with infinite thickness and homogeneous thermophysical properties. Similarly, the drug reservoir (77% *w/w* starch and 23% *w/w* glycerol) was modeled as a layer with effective thermophysical properties. Both the heater and the drug reservoir are encapsulated by cerebrospinal fluid, which was modeled as water for the sake of simplicity. Details of the thickness, heat capacity, heat conductivity, and mass density of each BEP layer are described in Supplementary Table 3. The temperature of the brain tissue at infinity was fixed at body temperature (37 °C), while the Mg heater temperature was set at 42 °C. At equilibrium, the temperature distribution within the brain tissue at heater temperature are shown in Fig. 3k as 2D plots. Along the depth direction in the brain, the temperature beneath the center of the BEP was plotted in Fig. 3l, and the shaded part represents the temperature range within which human tissues will not incur heat damage. Thus, Fig. 3l can be used to control the BEP temperature in order to avoid tissue damage.

***Modeling of DOX diffusion into the brain and induction heating.*** The diffusion of DOX into the brain was modeled as an isotropic diffusion process. Due to the significant temperature dependence of cell membrane permeability, we had to account for the temperature dependence of the diffusion coefficient. For the sake of simplicity, we adopted the following linearized diffusivity-permeation relation:

*D(T) = aP(T) + D_0_* $(6)$

where *D* is the diffusivity of DOX in brain, *T* is the temperature, *P* is the permeation of DOX in brain (which was approximated from previously published experimental data,^1{Mills, 2006 #53}{Mills, 2006 #53}^ and *a* and *D_0_* are constants to be determined. To express the temperature dependence of *P* explicitly, we fitted the reported experimental data^1^ using the modified Lorentzian asymmetric peak fitting method; this is plotted as the red solid curve in Supplementary Fig. 18a. The DOX profile was experimentally measured with the heater on or off after 30 min, 5 h, and 15 h. By fitting the measured DOX profiles, we obtained the brain diffusivity of DOX when *T* = 37 °C (*T_0_*; body temperature; reference temperature) and when *T* = 42 °C (Supplementary Fig. 18b and 17c).

*D*(*T* = 37 °C) = 3×10^-13^ m^2^ s^-1^ *D_0_* $(7)$

*D*(*T* = 42 °C) = 9×10^-12^ m^2^ s^-1^ *D_0_* $(8)$

From this, we calculated that *a* = 5.86×10^-8^ and *D_0_* = 1.13×10^-13^ m^2^ s^-1^ in the *D(T) ~ P(T)* relation. We assumed that the drug reservoir had a constant DOX concentration (c_0_) over time, and that the ambient/brain interface had no mass flux. Using this time-dependent diffusivity value, the brain DOX profile could be calculated for a particular brain temperature field.

COMSOL was used to calculate the brain temperature field achieved using the induction-heating module. To reduce the computational complexity of 3D induction heating, we simplified the model by reducing the detailed 3D structure of the Mg heater to a 2D structure (Supplementary Fig. 18d) by leveraging the axisymmetry of this problem. The entire BEP/brain system rested in an ambient environment at a room temperature of 20 °C. A 5-turn copper coil was placed above the BEP with an electric current of 360 A (frequency; 220 kHz, Easyheat, Ambrell, USA). The resistivity of the copper coil (*ρ*_c_), was linearized as a function of *T*:

$\rho_{c}=\rho_{0}\left[ 1+\alpha_{T}\left( T-T_{0} \right) \right]$ $(9)$

where *ρ*_0_ was the reference resistivity of copper (1.76×10^-8^ Ωm), *α_T_* was the temperature coefficient (0.0039 K^-1^), and *T_0_* = 20 °C (the reference temperature). Cooling water ran through the induction coil and this was expressed as:

$$Q_{0}=\frac{M_{t}C_{p}\left( T_{in}-T \right)}{2\pi rA} (10)$$

where *M_t_* is the inlet flow rate (15 kg min^-1^), *T_in_* is the inlet temperature (293 K), *C_p_* is the heat capacity of water (4187 J kg^-1^ K^-1^, *r* is the radial position of the coil, and *A* is the cross-sectional area of the coil. The coil had inner and outer radii of 20 mm and 25 mm, respectively, and its cross-section was a circular ring with an inner radius of 1.5 mm and a thickness of 1 mm.

When modeling the inductive heating, the whole domain was considered to be governed by Ampere’s law and heat transfer; this included the air, coil, BEP, and brain tissue. At the extremities of the air and brain domains, magnetic insulation boundary conditions were used to calculate the magnetic field. The temperatures in the depth direction were set at room temperature (20 °C) for the air and body temperature (37 °C) for the brain. Lateral heat flux was calculated using

*q* = *h*Δ*T* $(11)$

where the heat transfer coefficient (*h*) was 5 W m^-2^ K^-1^ for the air domain and 10 W m^-2^ K^-1^ for the brain domain.

For this inductive heating-diffusion problem, we assumed that the inductive heating achieved equilibrium much faster than the diffusion process. Thus, the coupled magnetic-temperature field was first solved using the frequency-stationary method; the resultant temperature field was then used in the diffusion model, which was solved using a time-dependent method in COMSOL. A contour plot of the temperature distribution in the whole system is shown in Fig. 3k, where the distance between the bottom of the coil and the surface of the brain was set to be 2 cm. The normalized DOX profile in the brain for the same coil-brain distance is provided in Supplementary Fig. 18e. The distance between the coil and the brain is a tunable parameter that could be used to control the heating power.

***Finite element modeling of 3D thermal profiles at various skull thickness.*** Finite element simulations were performed using COMSOL 5.2 to reveal the heater temperature in various conditions of skull thickness.

The brain tissue was simplified to be only represented by white matter, and surrounding environments are same as “***Finite element modeling of 3D thermal profiles at various heater temperatures***”***.*** Details of the thickness, heat capacity, heat conductivity, and mass density of each BEP layer are described in Supplementary Table 3. For the sake of simplicity, we use the same material properties for human, canine, and mouse skulls as listed in the Supplementary Table 4. Relative permittivity, density and electric conductivity is approximated by porcine and ovine skull measurements^2, 3^. Thermal conductivity is approximated by measurements on the shoulder blade of a pig, and sheep skull^4^. As shown in Supplementary Fig. 19, the elevated temperature of the heater is independent regardless of the skull thickness.

- 1. **Wireless temperature sensor.**

The wireless temperature sensor was designed as an LC oscillator made of Mg and PLGA (lactic acid:glycolic acid; 65:35) was used as the dielectric material because its glass transition temperature is ~39 °C, which is close to the body temperature. As the temperature increases above the body temperature, the glass transition changes the morphology of PLGA, resulting in a change in the dielectric permeability. This also changes the resonance frequency of the LC oscillator. Thus, a temperature change was detected by observing the change of the sensor resonance frequency. A conventional inductor coil was used as a reader coil to detect the resonance frequency change of the sensor wirelessly. Inductance coupling between the sensor and the reader coil occurs when they are close to each other and in parallel. As the resonance frequency of the sensor responds to the temperature increase, the resonance peak of the inductively coupled reader coil is also altered. Therefore, the BEP temperature change was wirelessly monitored by observing the resonance peak of the reader coil with a Network Analyzer (Agilent B1500A, Agilent Technologies, USA).

- 1. **Mouse subcutaneous GBM model.**

This study was approved by our Institutional Animal Care and Use Committee (IACUC; No. 14-0156-C1A3) and was performed in accordance with our IACUC guidelines and with the National Institute of Health Guide for the Care and Use of Laboratory Animals. The researchers who carried out surgery, managed the animal, and measured the tumor volume were blinded each other.

***Implantation of human glioblastoma cells into mice.*** Thirty-three 6-week-old female BALB/c nude mice weighing approximately 20–25 g were used in this study. Prior to tumor implantation, the animals were sedated with an intramuscular injection of 5 mg of a 3:1 combination of tiletamine hydrochloride and zolazepam (Zoletil; Virbac, Carros, France) and xylazine hydrochloride (Rompun 2%; Bayer Korea, Korea) per kg body weight. Then, U87-MG cells were subcutaneously transplanted (2×10^5^ cells/100 μL medium/mouse) into the thigh of the nude mice.

***Subcutaneous implantation of BEP.*** Fourteen days after implantation, human U87-MG tumors implanted in BALB/c nude mice had grown to a radius of ~4 mm. The tissues were exposed and resected, leaving ~ 30 mm^2^ of tumor. A BEP (12 mm in diameter) containing 0.69 mg DOX was then implanted onto the residual tumor (Supplementary Fig. 21). During the treatment period, the tumor volume was tracked by MR imaging every week. The tumor area in each MR image slice was measured and multiplied by the layer thickness (1 mm). Then the summation of the volume from each slice was regarded as a total tumor volume. Four different treatment groups were used to investigate the therapeutic effects of BEP: a control IV group received an intravascular injection of the equivalent amount of DOX, immediately after resection surgery; the Heating group received an implantation of BEP containing no DOX, with wireless mild-thermic actuation; the OST group received an implantation of BEP containing DOX, without mild-thermic actuation; and the OST+Heating group received an implantation of BEP containing DOX, with mild-thermic actuation. The groups receiving wireless heating were treated for 30 min daily for 14 days.

***MR image acquisition.*** The tail vein was catheterized after anesthesia with 1.5‑2% isoflurane/oxygen (*v/v*), and the animals were placed in a 9.4 T MR image scanner (Agilent Technologies, USA). Throughout each imaging session, the animals were wrapped in a warm water blanket and their oxygen saturation and heart rates were monitored. A millipede 1-ch coil was used for both radio frequency transmission and signal reception (Agilent Technologies, USA) and a fast spin echo sequence was used to produce a T2-weighted image. The measurement parameters were as follows: repetition time = 3000 ms; effective echo time = 30.82 ms; field-of-view = 20×35 mm; echo train length = 4; matrix = 256×256; slice thickness = 1.0 mm.

- 1. **Mouse brain GBM model.**

This study was approved by the Institutional Animal Care and Use Committee (IACUC; No. 14-0156-C1A3) and was performed in accordance with the IACUC guidelines and with the National Institute of Health Guide for the Care and Use of Laboratory Animals.

In this study, human glioblastoma cell line U87-MG which was obtained directly from ATCC (HTB-14) was used for GBM model. Before implantation, the cell line was re-authenticated using microsatellite profiling and routinely tested to exclude infection with mycoplasma.

For mouse GBM model, we examined 15 male BALB/c nude mice (mean weight, 20 ± 25 g). The mice were anesthetized with a mixture of zolazepam and xylazine and were placed in a stereotaxic device. Total 3×10^6^ cells were injected per mouse using a Hamilton syringe fitted with a 28-gauge needle, which was positioned by the stereotaxic device. The following coordinates with the stereotaxic guidance were used: AP -1.3 mm, ML +2.0 mm, and DV -1.0 mm. A week after cranial implantation, intracranial implantation of device was performed.

***Intracranial implantation of devices.*** A 2 mm diameter and 1 mm height gold heater was attached to the OST film of 3 mm diameter. The OST film contained 0.13 mg DOX. The control wafer containing 0.88 mg carmustine was also implanted. After fixing the mouse head position, we cut the skin of the head and drilled the mouse skull in a round shape for implantation of the patch. Devices were implanted on the tumor tissue, and then their positions were fixed using N-butyl cyanoacrylate (Supplementary Fig. 23c). During the treatment period, the tumor volume was tracked by MR imaging every week. Three different treatment groups were used to investigate the therapeutic effect in the brain microenvironment: The sham operating group received a physical surgery protocol and anesthesia like different groups but without implantation; the control wafer group received implantation of the control wafer containing carmustine without mild-thermic actuation; and the OST+Heating group received implantation of the gold heater and OST film containing DOX with mild-thermic actuation (30 min daily for 14 days).

***MR image acquisition****.* After anesthesia with 1.5/2% isoflurane/oxygen (v/v), the animals were placed in a 9.4 T MR image scanner (Agilent Technologies, USA). Throughout each imaging session, the animals were wrapped in a warm water blanket and their oxygen saturation and heart rates were monitored. A Rapid 1H surface Coil (RAPID Biomedical GmbH, Rimpar, Germany) was used for both radio frequency transmission and signal reception (Agilent Technologies, USA) and a fast spin echo sequence was used to produce a coronal T2-weighted image. The measurement parameters were as follows: repetition time = 3000 ms; effective echo time = 31.18 ms; field-of-view = 25×25 mm; echo train length = 4; matrix = 256×256; slice thickness = 1.0 mm.

***Mild-thermic actuation and therapeutic efficacy****.* The gold heater on the OST film provides sufficient heating (Supplementary Fig. 23f) for mild-thermic actuation (Δ*T* = 5 °C). Compared to the control, recurrence of brain tumor was suppressed (*p* = 0.0317; Supplementary Fig. 23g-j) in the ‘OST+heating’ group. The survival time was also prolonged in the ‘OST+Heating’ group compared to the control (*p* = 0.0011; Supplementary Fig. 23k).

- 1. **Canine GBM model.**

This study was approved by our Institutional Animal Care and Use Committee (IACUC; No. 14-0156-C2A3) and was performed in accordance with our IACUC guidelines and with the National Institute of Health Guide for the Care and Use of Laboratory Animals.

***Preparation of canine brain tumor fragments for implantation.*** J3T-1 cells were prepared in 100 mL serum-free RPMI medium and then subcutaneously transplanted (2×10^5^ cells/100 μL medium/mouse) into the shoulders of 6-week-old female BALB/c nude mice (Koatech, Korea). The mice were sacrificed 14 days after the injection, and tumors were extracted to make fragments of 20 mm in diameter, which were washed with phosphate-buffered saline (PBS) and suspended in 50% basement membrane matrix (Matrigel, BD Bioscience, USA) in an icebox. For each dog, 10 brain tumor fragments of 3–5 mm in diameter were prepared for implantation.

***Canine brain tumor implantation.*** 2 Adult male mongrel dogs per group (International Laboratory Animal Center, Korea) weighing approximately 10–12 kg were used in this study. Each dog was treated with immunosuppressive agents before tumor implantation and for the duration of the study. For immunosuppression, the dogs were treated for 7 days with an oil-based cyclosporine formulation (Sandimmune; Novartis Pharm., Switzerland) (400 mg orally, twice daily). In addition, 50 mg of azathioprine (Azaprine Tab., Korea United Pharm. Inc., Korea) and 10 mg of prednisolone (Solondo Tab., YuhanMedica, Korea) were mixed with normal saline and then administered orally twice daily. This regimen was previously used in an intraparenchymal and cavernous sinus tumor model in canines^5, 6^.

After 7 days of immunosuppressive agent administration, tumor implantation was performed in the dogs (Supplementary Fig. 24). The animals were first sedated with an intravenous injection of 5 mg of a 1:1 combination of tiletamine hydrochloride and zolazepam (Zoletil; Virbac, Carros, France) and xylazine hydrochloride (Rompun 2%; Bayer Korea, Korea) per kg of body weight. The dogs were then intubated and placed on a ventilator. Anesthesia was maintained with isoflurane at a concentration of 0.5–3%, depending on the vital signs observed during surgery.

The scalp was shaved, scrubbed, and draped in an aseptic manner prior to making a scalp incision over the right frontoparietal region. The muscle was split with cautery and retracted. The underlying cranium was exposed, and a craniotomy was performed to create a burr hole (0.3–0.5 cm in diameter) using a handpiece drill (STRONG 204; Saeshin Precision Co., Ltd., Korea). After confirming the dural layer from the base of hole, the prepared tumor fragments were implanted in the right frontal lobe of each dog using an 18-gauge spinal needle. Bleeding from the procedure was controlled using bone wax (ETHW31G, Ethicon, USA) or *N*-butyl cyanoacrylate (Histoacryl, Braun, Germany). The overlaying muscle and skin were then closed using a 3-0 Vicryl suture and a 4-0 nylon suture.

All dogs were given intramuscular injections of cephazolin (Cefazolin; Chong Kun Dang Pharm. Co., Korea) to prevent infection, and surgical sites were swabbed with povidone-iodine was applied onto the surgical site for 7 days. Leukocyte and C-reactive protein counts were measured routinely to detect potential infections or signs of sepsis.

***Intracranial bioresorbable electronic patch (BEP) implantation.*** Seven to fourteen days after tumor implantation, canine brain tumors had grown to 1–2 cm in the longest dimension. Prior to surgery, anesthesia was induced as described above, and then a scalp incision was widely made over the right frontoparietal region along the previous incision site, under aseptic conditions. When the underlying cranium was exposed, a craniectomy was performed to establish the surgical view at the location of the tumor around the previous craniotomy site using a handpiece drill. The remaining bone was removed using a surgical elevator, and the dura was carefully dissected using micro scissors to expose the brain parenchyma. After careful removal of the brain parenchyma using bipolar forceps, the location of the tumor was matched with the magnetic resonance (MR) image, and finally the tumor was exposed. Tumor tissues were resected almost entirely, leaving microscopic residual tumor tissues (Supplementary Fig. 25). Then, the BEP (12 mm in diameter, containing 1 mg DOX) or control wafer (containing 2.38 mg carmustine) was positioned to cover the residual tumor sufficiently, and fixed with surgical glue (*N*-butyl cyanoacrylate; Histoacryl, Braun, Germany) (Supplementary Fig. 24). The muscles and skin were then sutured as described above.

***MR image acquisition.*** MR imaging was performed using a 3.0 T MR imaging system (Magnetom Trio; Siemens Medical Solutions, Germany) with a human head coil. Prior to MR imaging, anesthesia was induced as described above. All MR imaging examinations were performed with the dogs in the supine position and included the entire brain.

The brain imaging sequences included axial turbo-spin echo T2-weighted images (T2WI), axial gradient-echo T1-weighted images (T1WI), and axial contrast-enhanced (CE) T1WI. After routine localization images were obtained, T2WI (repetition time/echo time, 5160 ms/91 ms; flip angle 131°, section thickness, 5 mm; matrix, 640×290) and T1WI (repetition time/echo time, 990 ms/9.8 ms; flip angle 70°, section thickness, 1.5 mm; matrix, 384×212) were acquired. Subsequently, CE T1WI was acquired after an intravenous injection of 0.2 mL/kg godoteric acid (Dotarem; Guerbet, France) via the cephalic vein.

- 1. **Materials.**

All of the materials were used as purchased, unless stated otherwise. Starch from corn (unmodified waxy corn starch of essentially pure amylopectin), sodium periodate (NaIO_4_), PLA (M_w_ ~ 260,000), PLGA (lactic acid:glycolic acid; 65:35; M_w_ 40,000‑75,000), PBS, polyimide precursor solution (poly(pyromellitic dianhydride-co-4,4′-oxydianiline), amic acid solution (electronic grade), and 1-methyl-2-pyrrolidinone (> 99%) were purchased from Sigma Aldrich (USA). Glycerol was purchased from Samchun Chemical (Korea), DOX∙HCl was purchased from Ildong Pharm (Korea), and polydimethylsiloxane base with curing agent (Sylgard 184) was purchased from Dow Corning (USA).

For device fabrication, the thermal evaporating source of Mg (> 99.5%) was purchased from Taewon Scientific Co. (Korea), the sputter target of ZnO (99.99%) was purchased from Thifine (Korea), the Si wafer was purchased from 4Science (Korea), poly(methylmethacrylate) (PMMA A11) was purchased from MicroChem (USA), and positive photoresist S1805, AZ5214, and AZ4620 were purchased from AZ Electronics Materials (USA).

- 1. **Synthesis of OST.**

NaIO_4_ was used as the reducing agent for the synthesis of OST. First, 2.14 g NaIO_4_ was dissolved in 250 mL water before adding various amounts of starch. Next, hydrochloric acid was used to adjust the pH to 3‑4. The solution was strongly stirred overnight at 40 °C, and the resultant OST was filtered and washed 3 times with 0 °C deionized water. The final OST product was dried for 24 h at 40 °C under a vacuum.

- 1. **Fabrication of starch and OST films containing DOX.**

To produce a normal starch patch containing DOX, 1.5 g starch powder, 50 mg DOX, and 0.45 g glycerol were dissolved in 40 g water at 80 °C. The mixture was stirred for 30 min until a clear solution was obtained. The solution was then poured into a petri dish (90-mm diameter) and dried at 65 °C with 80% humidity for 48 h.

To produce an OST film containing DOX, 1.5 g OST powder and 50 mg DOX were dissolved in deionized water and strongly stirred for 24 h at 80 °C to form the imine linkage between OST and DOX. Glycerol (0.45 g) was then added to the mixture and after 1 h, the solution was poured into a petri dish and dried, as described above.

- 1. **Flexibility of OST films containing various glycerol concentrations.**

Dried OST films fabricated using a range of glycerol concentrations were cut into rectangles of 5×50 mm^2^. Each end of the patch was fixed to a digital force gauge (Series 4, Mark-10, USA) and stretched at a speed of 20 mm/min until it fractured. The elastic modulus was calculated from the slope of stress-strain curve and the maximum length was achieved at the rupture point.

- 1. **Adhesion force between tissue and OST films containing various concentrations of reducing agent.**

Dried OST films fabricated using different levels of aldehyde were cut into rectangles of 10×20 mm^2^. One side of each film was attached to bovine muscle as a representative biological tissue; this was used because the adhesion force between the patch and brain tissue was larger than the internal coherence of the brain tissue. The other side of the film was fixed to a digital force gauge (Series 4, Mark-10, USA) and a shear force was applied at a speed of 10 mm/min until the film was detached from the muscle. The shear stress at failure was calculated.

- 1. **DOX release profiles from starch and OST *in vitro.***

The DOX release profiles were determined by measuring the absorbance of the solution at 480 nm using SpectraMax M3 (Molecular Devices, USA). Each patch was the same size (20×20 mm^2^) and contained 3.387 mg DOX. Each patch was placed in 50 mL PBS and the DOX concentration was measured over time (n = 4). The 500 μL of PBS was extracted from the 50 mL solution for the measurement every day, and the entire PBS was replaced every 2 days to mimic *in vivo* cerebrospinal circulation^7^. The concentration was divided by the decreased volume in the concentration calculation. The released DOX was determined using a standard curve of DOX concentration versus absorbance, and cumulative release was calculated by integrating these measurements.

- 1. **Measurement of DOX diffusion using fluorescence microscopy.**

The implanted BEP was retrieved and the tumor tissue was extracted. Tissue blocks of the entire tumor were cut, embedded in optimal cutting temperature compound, and stored at -80 °C. Ten 10 μm serial frozen sections were obtained and examined using fluorescence microscope (DM5500 B, Leica, Germany) and camera (DFC365 FX, Leica, Germany) with filter sets for DOX (excitation/emission: 488/520 nm). All DOX diffusion profiles are measured by this method.

- 1. **Wireless RF heating performance of the heater.**

The performance of heaters of different sizes was evaluated using an infrared camera (i5, FLIR, USA). The patches were heated by 360 A coils with different distances. When the elevated temperature reached the maximum point and converged, the temperature was measured five times/s by the infrared camera (i5, FLIR, USA).

- 1. **DOX diffusion into tumor cells.**

*In vivo* DOX diffusion into tumor cells was monitored by fluorescence imaging. A BEP containing 0.69 mg of DOX was implanted subcutaneously in mice. After each implantation period, the BEP was detached from the tissue, which was then removed for DOX assessment. A 30-min pulse of mild-thermic actuation (ΔT = 5 °C) was used in the relevant experimental groups. The tissue diffusion profiles of DOX were analyzed using LAS AF Lite (LEICA, USA) with a quadrangle block, starting from the BEP-tissue interface and moving into the tissue. The average fluorescence intensities at each distance were transformed into DOX concentrations using calibration. To obtain this calibration, excised fresh tumors (3×3×2 mm^3^) were incubated with 34 µM DOX in PBS for 72 h. The tumors were then removed from the DOX solution and cryosectioned. The remaining DOX concentration was measured using SpectraMax M3 (Molecular Devices, USA), as previously described. The amount of DOX taken up by the tumors was then calculated by subtraction, and equated with the fluorescent intensity of the tumor tissue. The measured concentration-intensity relationship was also used to calibrate the diffusion profile *ex vivo*.

- 1. **Neurological tests**

*In vivo* electroencephalography surgery were performed as previously described^8, 9^. This is for detecting whether a severe degree of seizure may occur due to the implanted BEP. For electroencephalographic monitoring, the mice were subjected to electroencephalographic surgery 3 days before the implantation of the BEP. For surgery, the animals were anesthetized by intraperitoneal injection of 1% ketamine (30 mg/kg) and xylazine hydrochloride (4 mg/kg). The surgery was performed using a stereotaxic apparatus (Kopf Instruments, Tujunga, CA, USA). Electroencephalograms were obtained with tungsten electrodes (0.005 inch, 2 MΩ), which were positioned onto the right hemisphere at AP −1.8 mm, L 2.1 mm, and DV 0.8–1.0 mm (cortex) from the bregma with grounding over the cerebellum. The electrical activities were recorded after amplification (×1200), bandpass-filtering from 0.1 to 70 Hz, and digitization at a 400-Hz sampling rate (AS 40) with a digital electroencephalography system (Comet XL; Astro-Med, Inc., Warwick, RI, USA). Electrophysiological data were analyzed offline using PSG Twin 4.2 (Astro-Med, Inc.). Electroencephalographic signals in the mice were continuously recorded for 2 weeks. The epileptologist interpreted the intracranial electroencephalogram (iEEG) based on the occurrence of rhythmic burst discharges.

Rotarod performance of mice was assessed as previously described^10^ for verifying the indirect influence of the BEP implanted adjacent to the motor cortex, such as potential physical and/or chemical damages caused by implantation surgery, intracranial pressure change, and anti-tumor drug release. The accelerating Rota-Rod (San Diego Instruments, San Diego, CA, USA) set to linearly increase the speed from 4 to 40 rpm over 3 min. At baseline (pre), mice were trained on three consecutive days for three trials per day with a rest period of approximately 30 min between trials. The trial was terminated if the animal fell off the rungs or gripped the device and spun around for two consecutive revolutions. At each test day, mice were tested for three trials, and the mean latencies to fall were used for statistical analyses.

**Supplementary Figures**


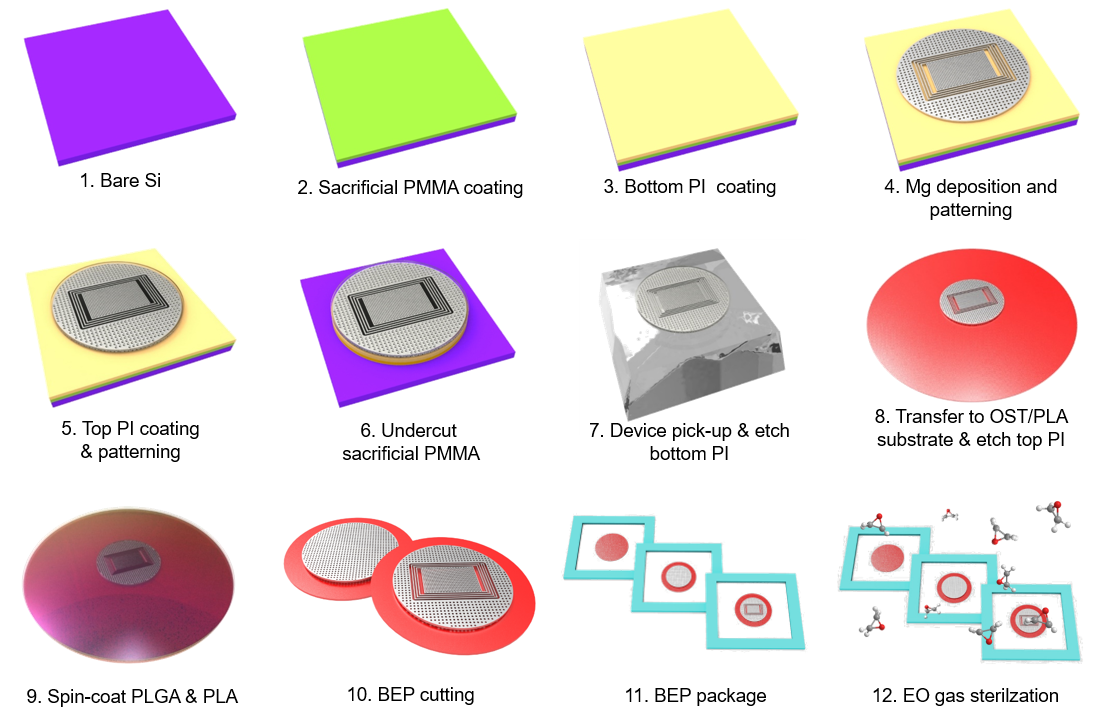


**Supplementary Figure 1**

Schematic illustration of the BEP fabrication process.


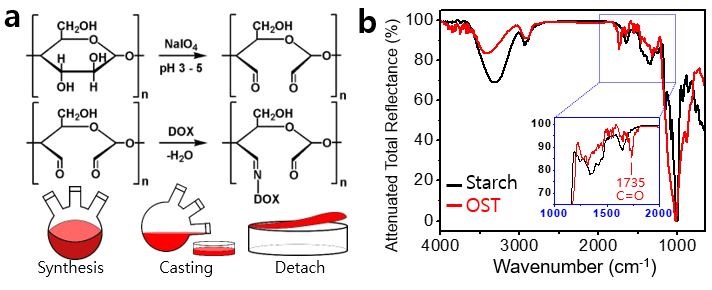


**Supplementary Figure 2**

(**a**) Synthesis and fabrication of the OST film. (**b**) Infrared spectroscopy analysis of unmodified starch and OST.


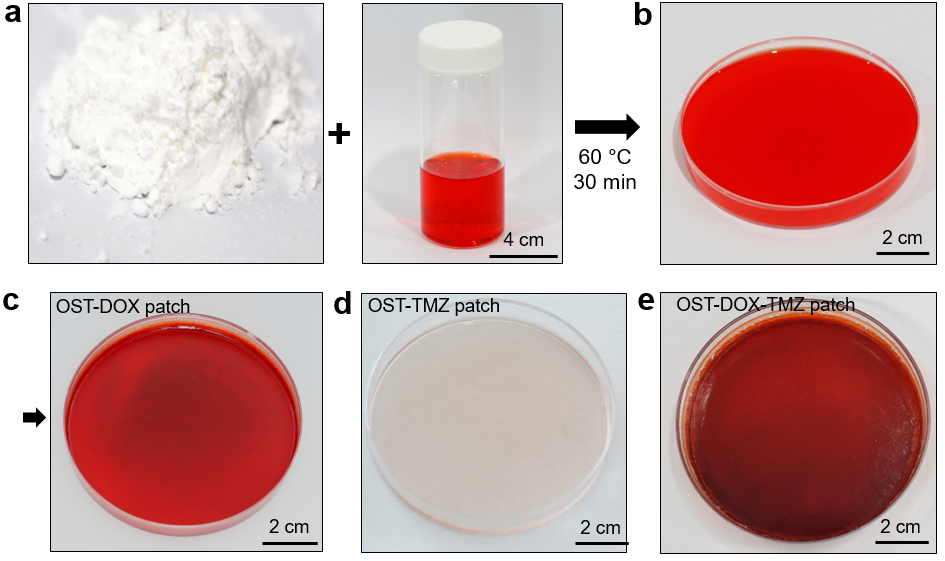


**Supplementary Figure 3**

Fabrication of the OST film. (**a**) Image of the waxy corn starch powder (left) and DOX solution (right). (**b**) Mixed solution molded in the 90 mm petri dish after heating at 60 °C for 30 min. (**c**) OST-DOX patch dried in the petri dish for 48 hours. (**d**) OST patch containing 20 mg temozolomide (TMZ). (**e**) OST patch containing both 20 mg of DOX and 20 mg of TMZ.


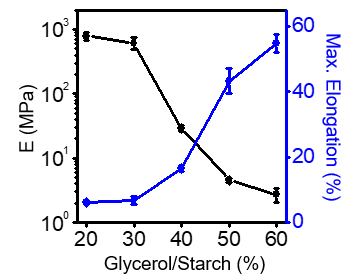


**Supplementary Figure 4**

Elastic modulus and maximum elongation of the starch film as a function of the glycerol concentration.


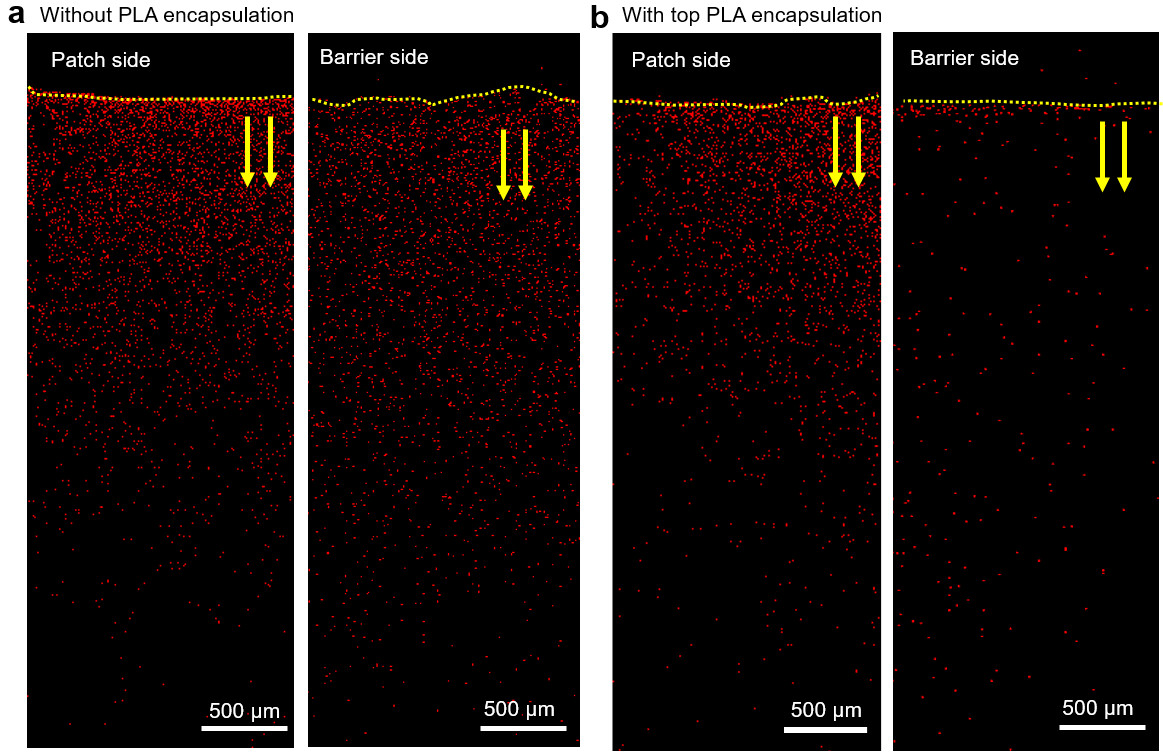


**Supplementary Figure 5**

Fluorescent images of DOX in the hydrogel that exhibit the amount of drug diffusion from the patch side (left) and the barrier side (right) of the BEP to the hydrogel (agar, 2 wt% in water) at 37 °C. (**a**) BEP without the top PLA encapsulation and (**b**) with the top PLA encapsulation.


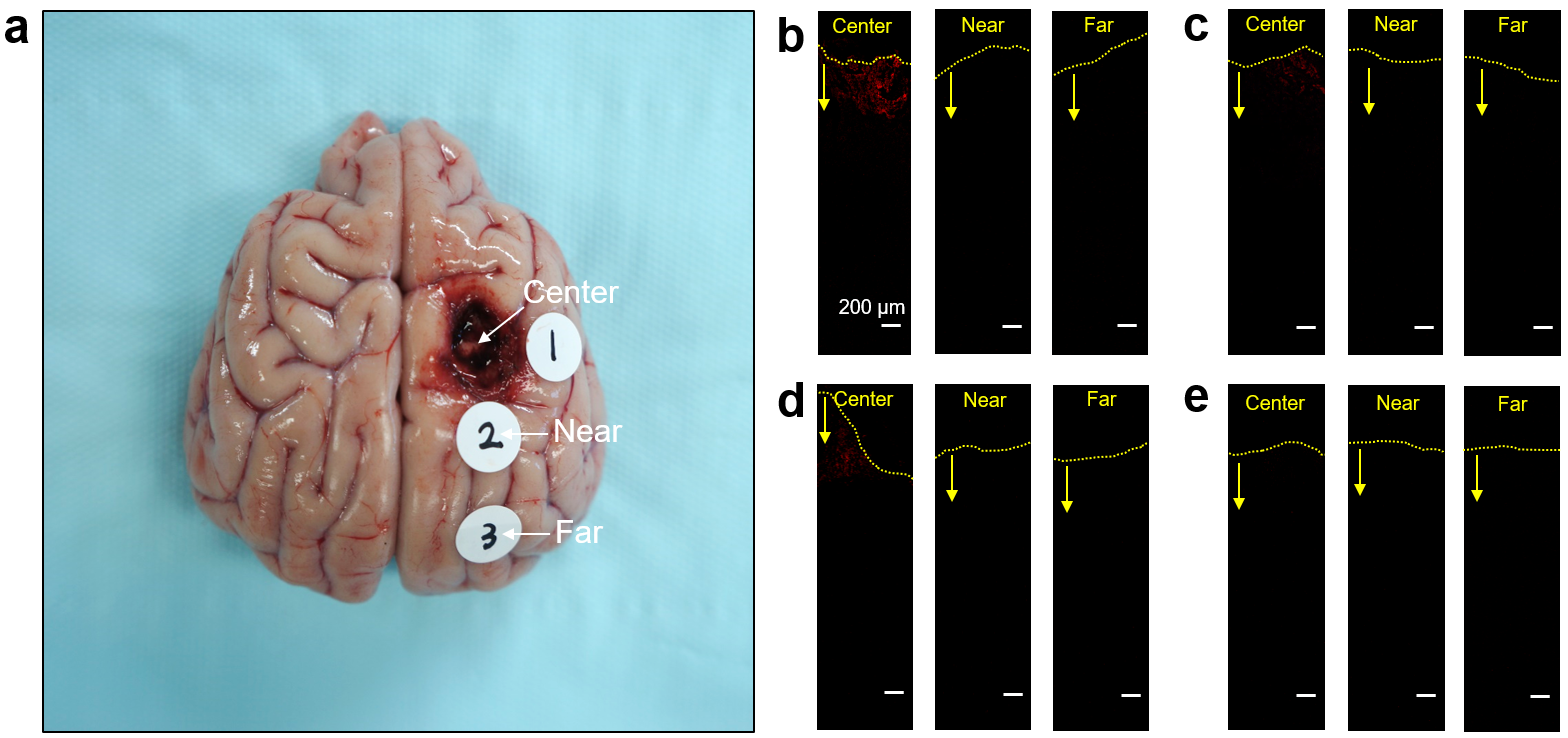


**Supplementary Figure 6**

Imaging of DOX at three different sites (1: center of the implantation site (the parietal lobe), 2: near the implantation site (the parietal lobe), 3: far from the implantation site (the occipital lobe)) in the canine brain at various time points. (**a**) Gross image of the canine brain at day 1 after the BEP implantation. Fluorescence imaging of DOX at three different sites in the canine brain at (**b**) 1 day, (**c**) 2 weeks, (**d**) 4 weeks, (**e**) 6 weeks after the BEP implantation.


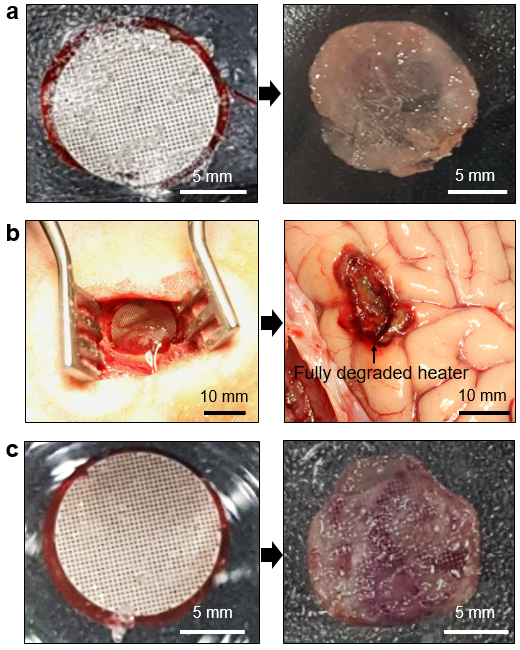


**Supplementary Figure 7**

The biodegradation of the wireless heater at different time points (left: day 0, right: day 14). Optical images of the biodegradable heater in (**a**) 37 °C PBS, (**b**) canine brain, and (**c**) 42 °C PBS.


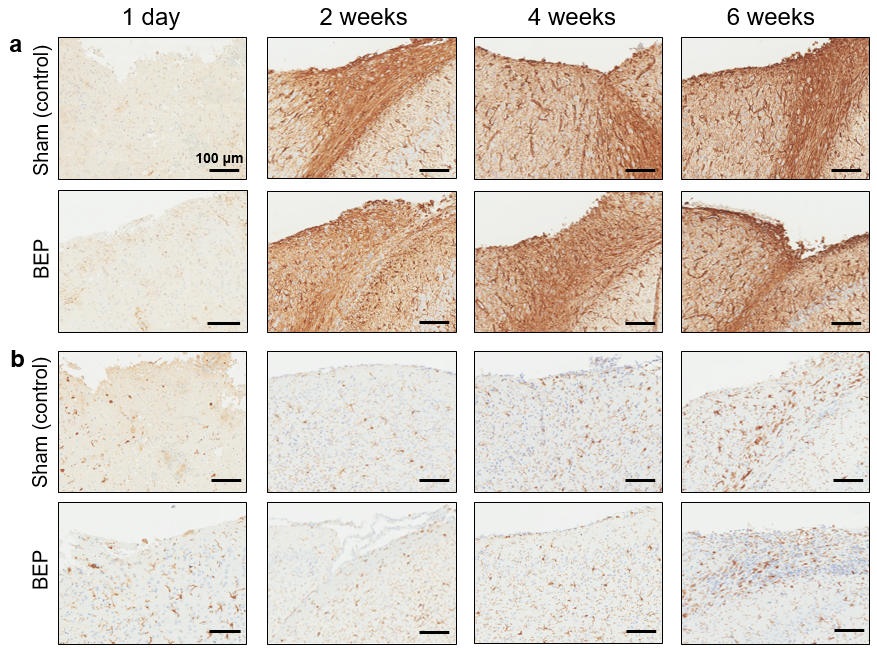


**Supplementary Figure 8**

(**a**) Confocal fluorescence microscopy images of the GFAP expression in the tissue slices from the implantation site in BALB/c nude mice groups (top for sham and bottom for BEP group) at different time points (1 day, 2 weeks, 4 weeks, and 6 weeks). (**b**) Confocal fluorescence microscopy images of the Iba-1 expression in the tissue slices from the implantation site in BALB/c nude mice groups. Other conditions are same with (**a**).


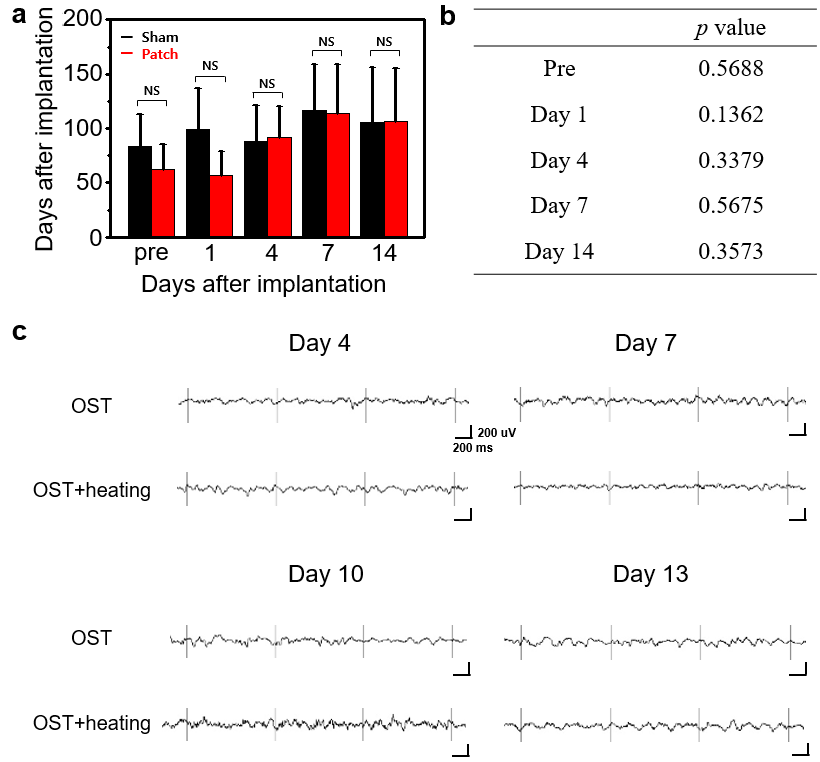


**Supplementary Figure 9**

Neurological test result to examine biocompatibility of the BEP. (**a**) Rotarod retention time of the sham operating group (black line) and the BEP implantation group (red line) at various time points after the surgery. Error bars represent the standard error of the mean value. n = 5, 6 for the sham and BEP implantation group. (NS; *p* > 0.05 by paired t-test) (**b**) *p*-values of the rotarod retention time in the sham and BEP implantation group at different time points, which are calculated by the paired t-test. (**c**) Intracranial electroencephalograms (iEEGs) of the sham operating group and the BEP implantation group at different time points.


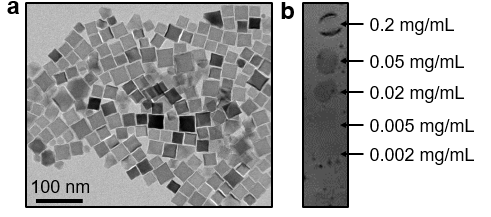


**Supplementary Figure 10**

Ferrimagnetic iron oxide nanocubes (FION) as the magnetic resonance (MR) contrast agent for intracranial bioresorption monitoring*.*

(**a**) Transmission electron microscope image of FION. (**b**) FION concentrations versus contrast values.


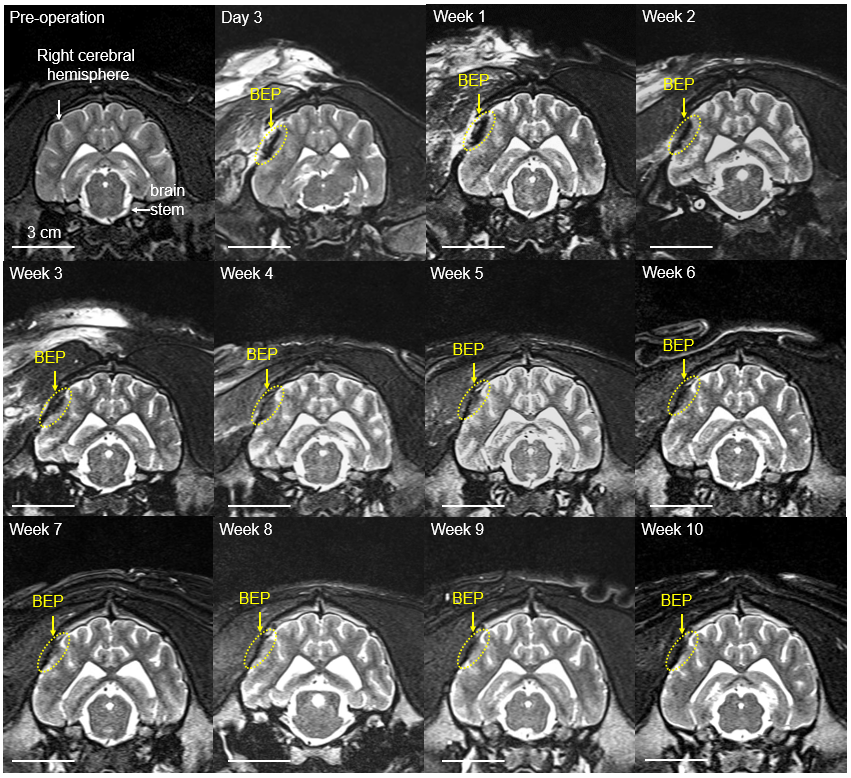


**Supplementary Figure 11**

MR images of the implanted BEP with the FION contrast agent at various time points. The device was implanted on the canine brain surface.


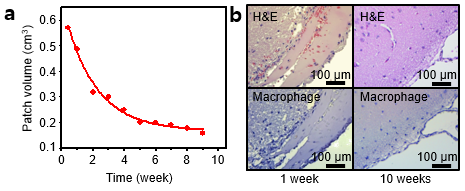


**Supplementary Figure 12**

(**a**) *In vivo* time-dependent patch volume change in the brain. (**b**) Histology images of tissues near the implantation site stained with hematoxylin and eosin (H&E; top) and macrophage antibody (bottom) after 1 week (left) and 10 weeks (right).


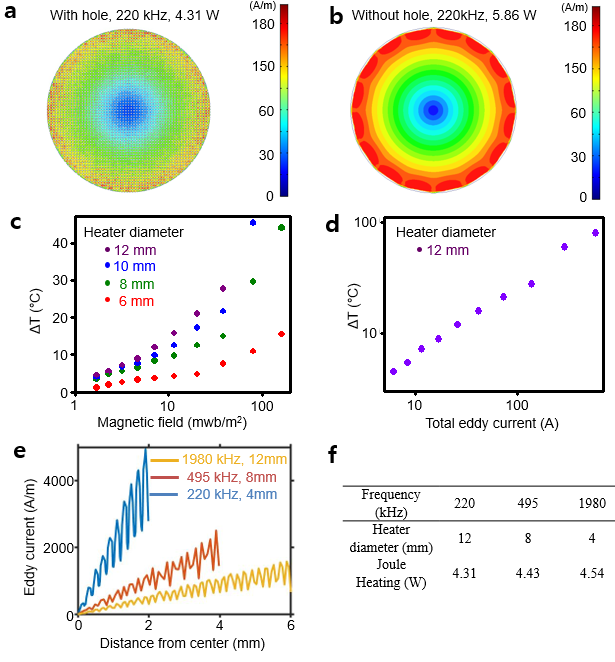


**Supplementary Figure 13**

Theoretical analysis and HFSS simulation of the wireless heater under different mild-thermic actuation conditions. 2-D Contour plot of the eddy current distribution of the BEP (**a**) with and (**b**) without the hole array under the optimized frequency. (**c**) Temperature increase by the wireless heater of various diameters as a function of the external magnetic field generated by the transmission coil. (**d**) Temperature increase by the wireless heater of 12 mm diameter as a function of the total eddy current. (**e**) 1-D eddy current from the center to the edge of the BEP with different frequencies and heater sizes. (**f**) Heat generation of the BEP with different frequencies and different heater sizes.


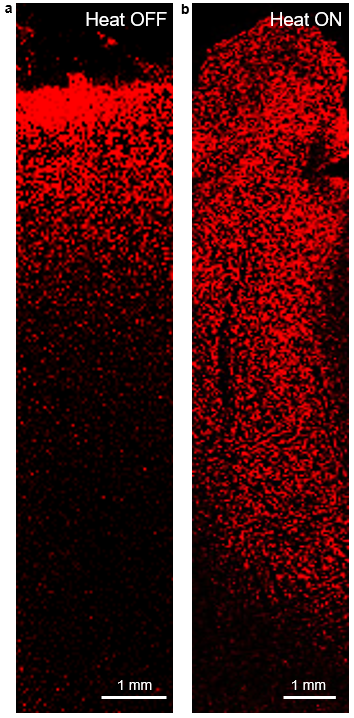


**Supplementary Figure 14**

Drug diffusion experiment over the extended period of time *ex vivo*. (**a**) DOX diffusion at 37 °C (left) and (**b**) 42 °C (right) for 48 h in the canine brain *ex vivo*.


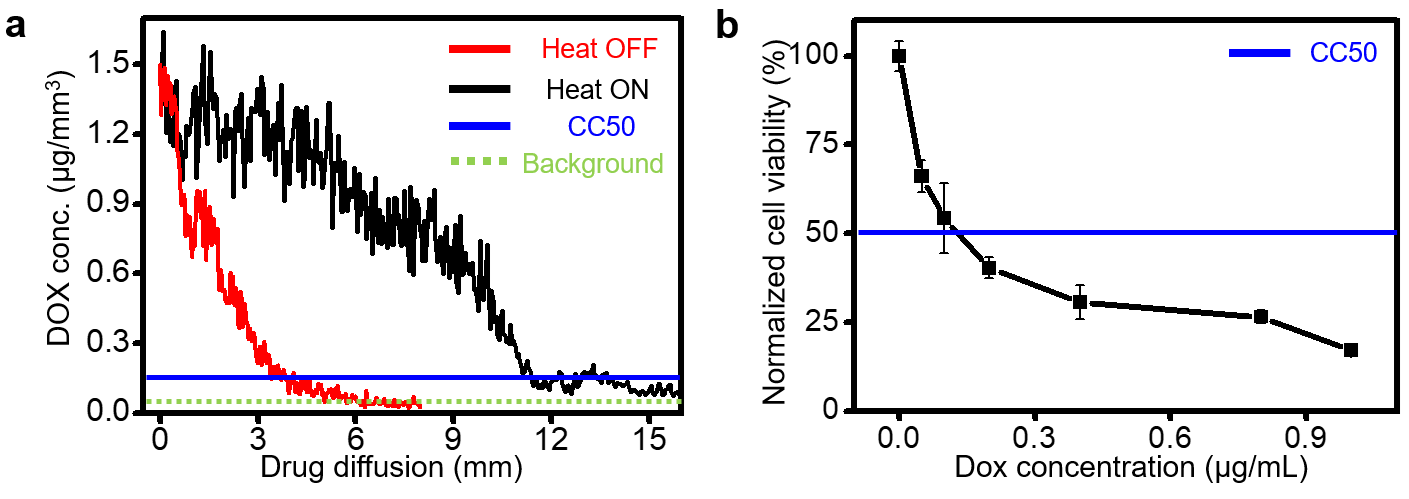


**Supplementary Figure 15**

DOX concentration measurement over the extended period of time *ex vivo* in the canine brain. (**a**) Distance dependent DOX concentration under the normal (37 °C; red) and mild-thermic actuation (42 °C; black) condition for 48 h in the canine brain *ex vivo*. (**b**) Cytotoxic concentration 50 (CC50) experiment of U87-MG in response to DOX.


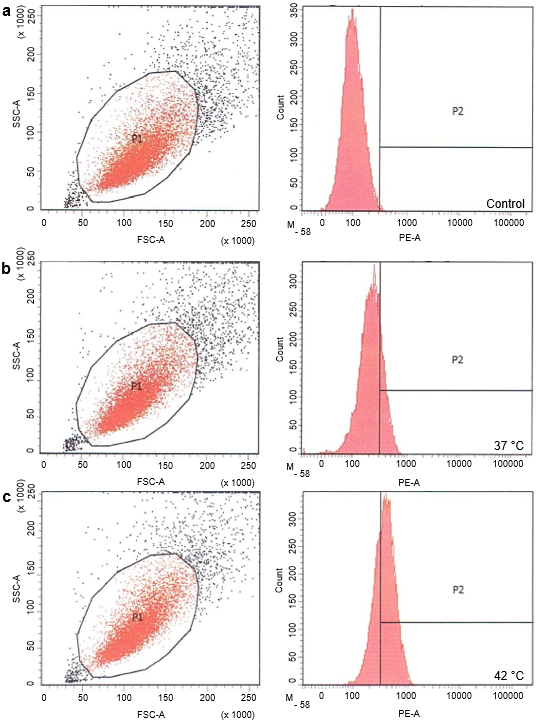


**Supplementary Figure 16**

Number of stained cells at different temperatures measured by flow cytometry. The left graphs show the tendency of Side-Scattered light Area (SSC-A) to Forward-Scattered light Area (FSC-A), and the right graphs show number of cells per Phycoerythrin area (PE-A). Cells are measured without DOX exposure for 1 hour a) negative control, and after exposure to the DOX solution for 1 hour at b) 37 and c) 42 °C.


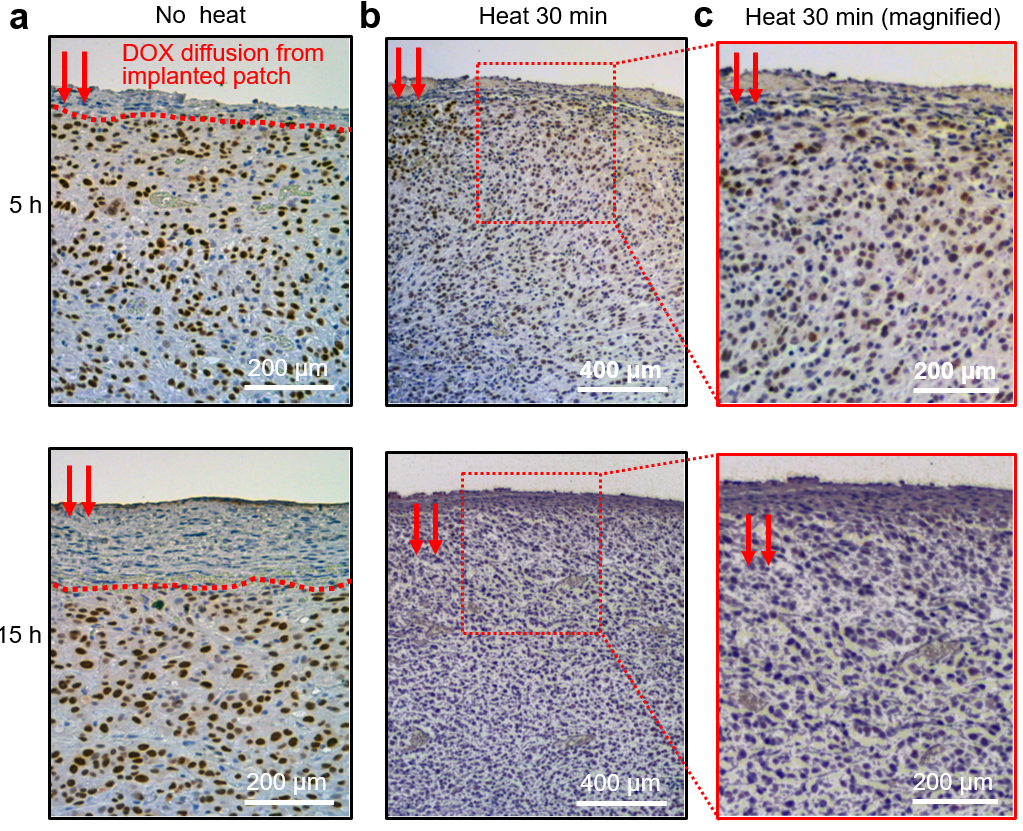


**Supplementary Figure 17**

Effect of DOX on survivin expression in U87-MG tumors at the indicated time-points. Survivin expression (**a**) without and (**b**) with heating (ΔT = 5 °C). (**c**) Magnified image of (**b**).


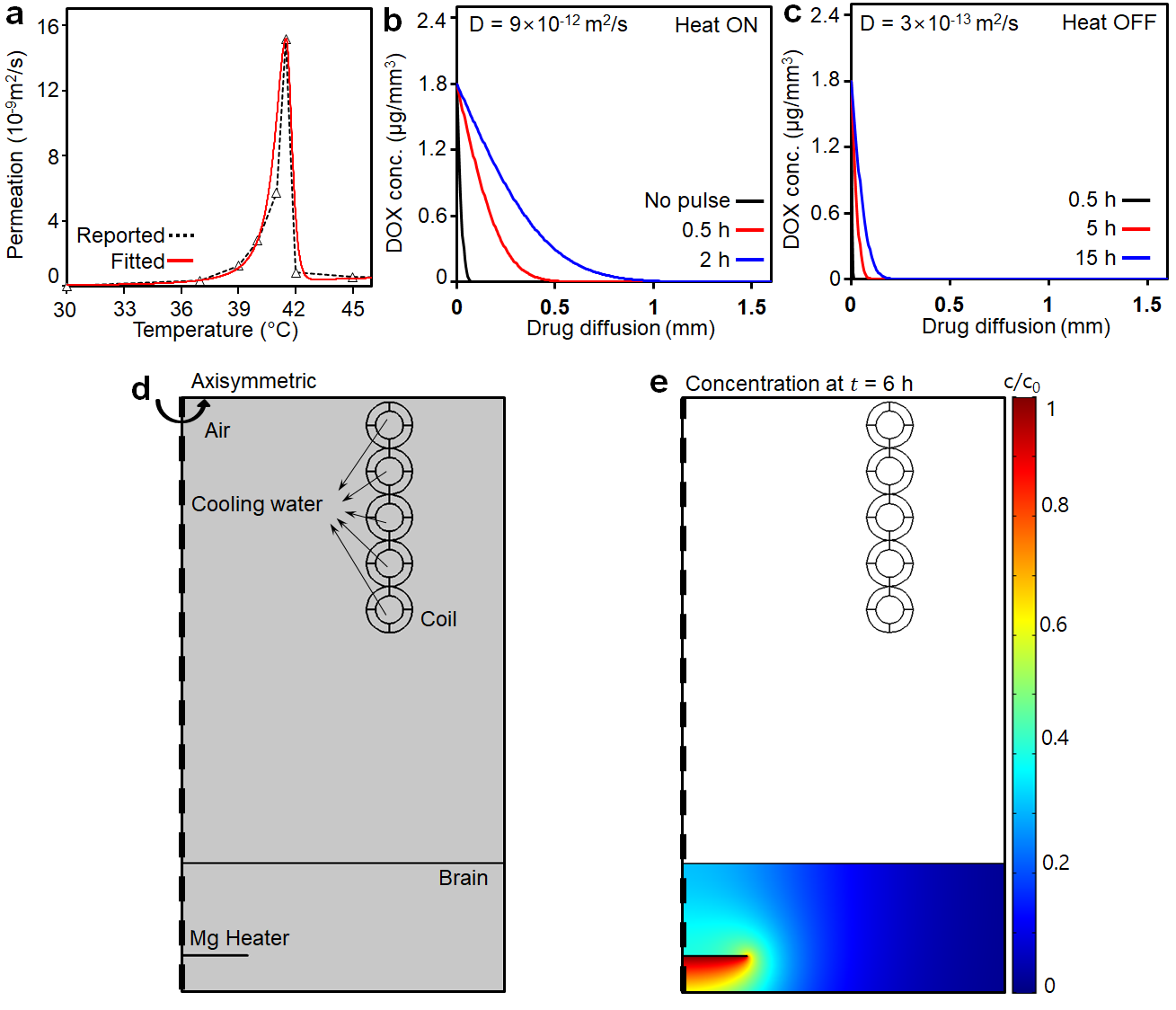


**Supplementary Figure 18**

Numerical simulations of drug diffusion in brain tissues as a function of temperature.

(**a**) Plot of previously reported data and corresponding fitting of DOX permeation versus temperature in the lipid bilayer. Fitted plot of the DOX concentration as a function of distance from the interface between the BEP and brain, (**b**) without and (**c**) with heating. (**d**) Simulation input and (**e**) the normalized DOX profile in brain.


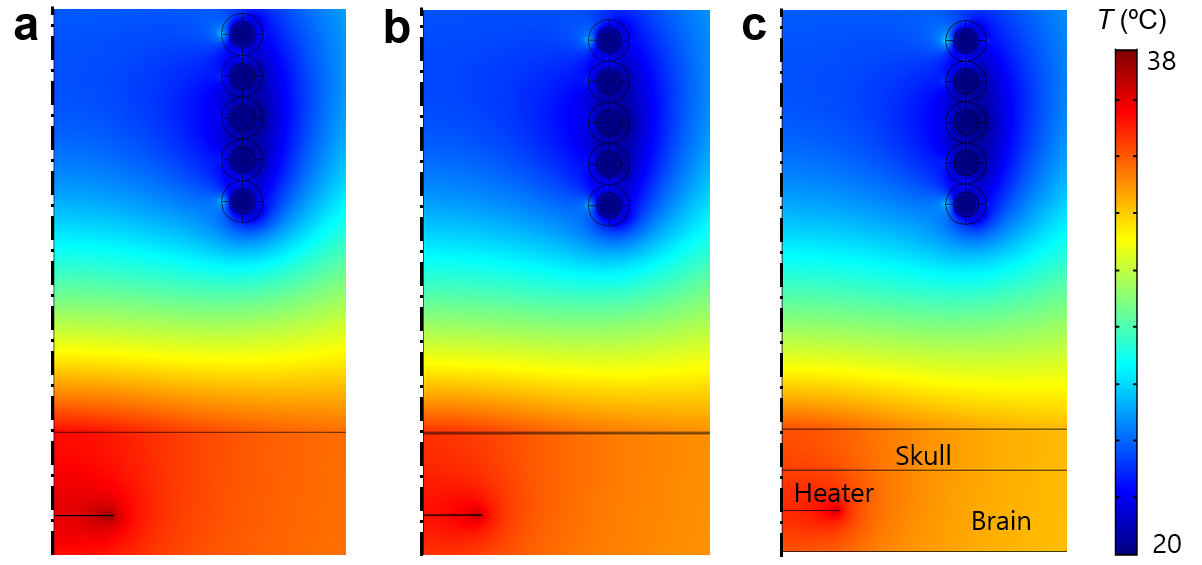


**Supplementary Figure 19**

The 3D FEM simulation results of contour plots depending on the different skull thickness. (**a**) No skull, (**b**) 0.15 mm thickness of skull (similar to mouse skull thickness), and (**c**) 5 mm thickness of skull (similar to human skull thickness).


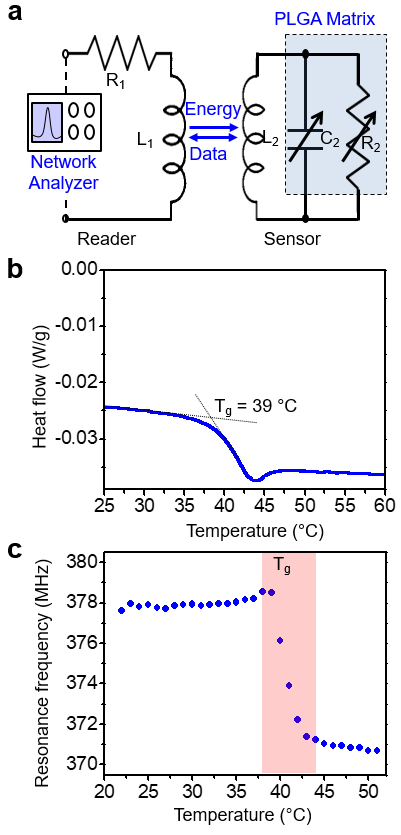


**Supplementary Figure 20**

Schematic illustration and characterization of the wireless temperature sensor.

(**a**) Circuit diagram of the wireless temperature sensor. (**b**) Differential scanning calorimetry curve around the glass transition temperature of poly(lactic-co-glycolic acid). (**c**) Resonance frequency change of the sensor as a function of temperature.

**
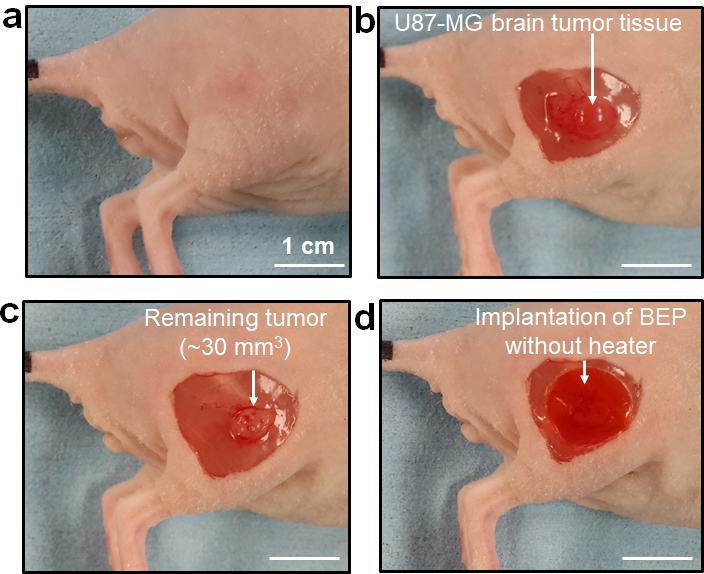
**

**Supplementary Figure 21**

Surgical process for the mouse brain tumor model.

(**a**) Optical camera image of an anesthetized mouse, (**b**) after resection of the mouse skin for brain tumor resection, (**c**) after the tumor resection surgery, and (**d**) after implantation of the BEP on the residual tumor.

**
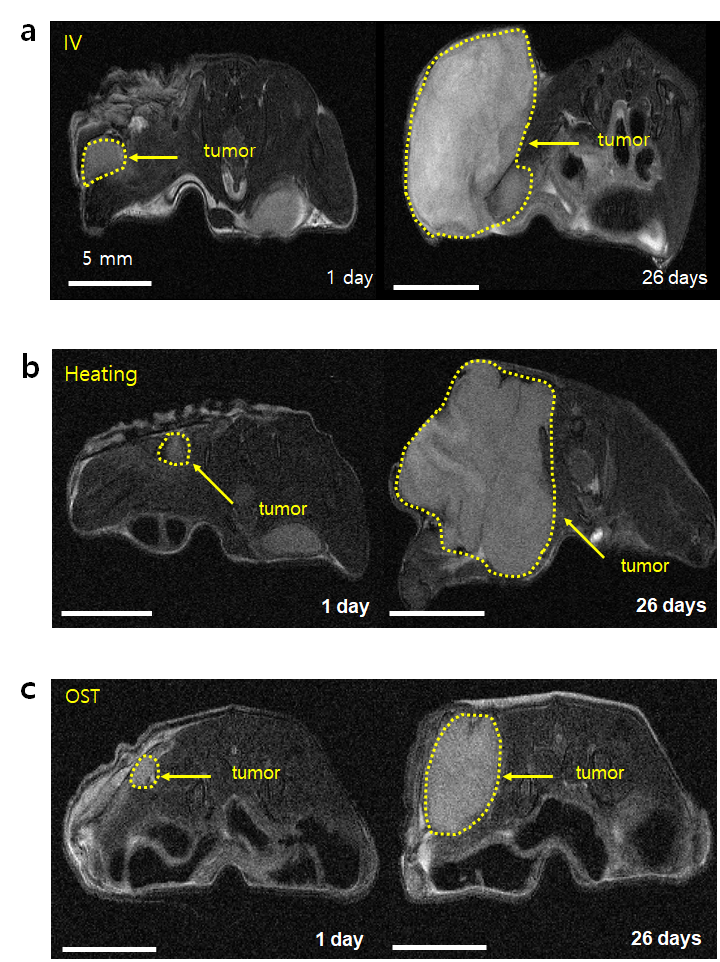
**

**Supplementary Figure 22**

MR images of the (**a**) IV group, (**b**) Heating group (no DOX), and (**c**) OST group (no heating) at the indicated time-points after surgery.


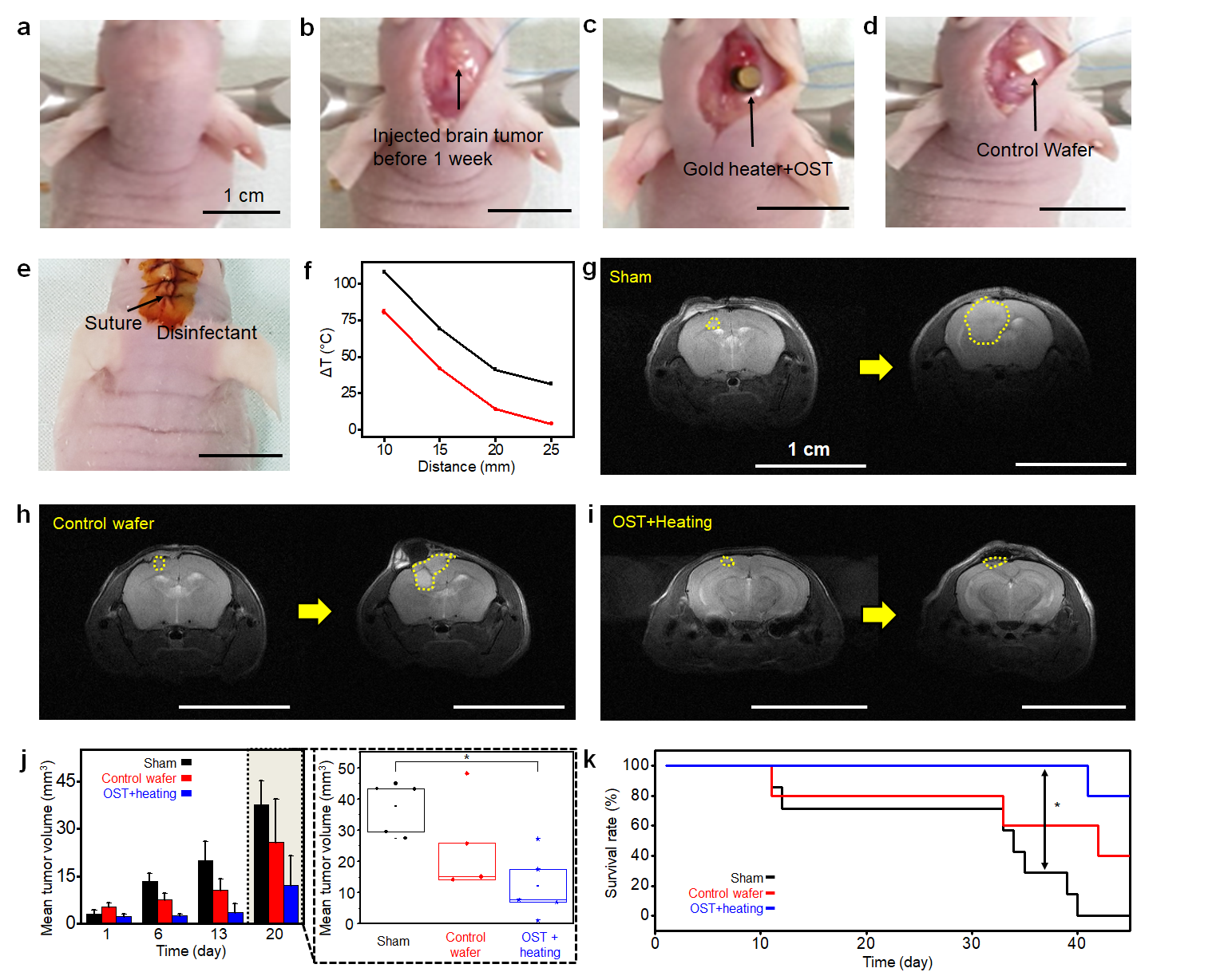


**Supplementary Figure 23**

Surgical process and therapeutic efficacy of the BEP in the mouse brain tumor model. (**a**) Fixation to the stereotaxic equipment and (**b**) incision of the skin of the mouse brain tumor model for neurosurgery. (**c**) Implantation of the miniaturized device composed of a gold heater and the OST patch and (**d**) a control wafer to the brain. (**e**) Skin suturing and disinfectant treatment. (**f**) Distance-dependent temperature changes of gold heater. Representative coronal T2-weighted MR images of the (**g**) ‘Sham’ group, (**h**) ‘control wafer’ group, and **i**. ‘OST+heating’ group at 1 day (left) and 2 weeks (right) after the surgery. (**j**) Time-dependent mean tumor volumes of the indicated groups (left) and box-and-whisker plots of tumor volumes at 20 days after the surgery (right). n = 7, 5, 5 for Sham, control wafer, and OST+heating group, respectively. Line: median Box: 25^th^ to 75^th^ percentiles, Whisker: min to max, **p* < 0.05 by Man-Whitney U-test with Bonferroni correction. (**k**) Kaplan-Meier survival rate plots of the indicated group. **p* < 0.05 by log-rank test with Bonferroni correction.


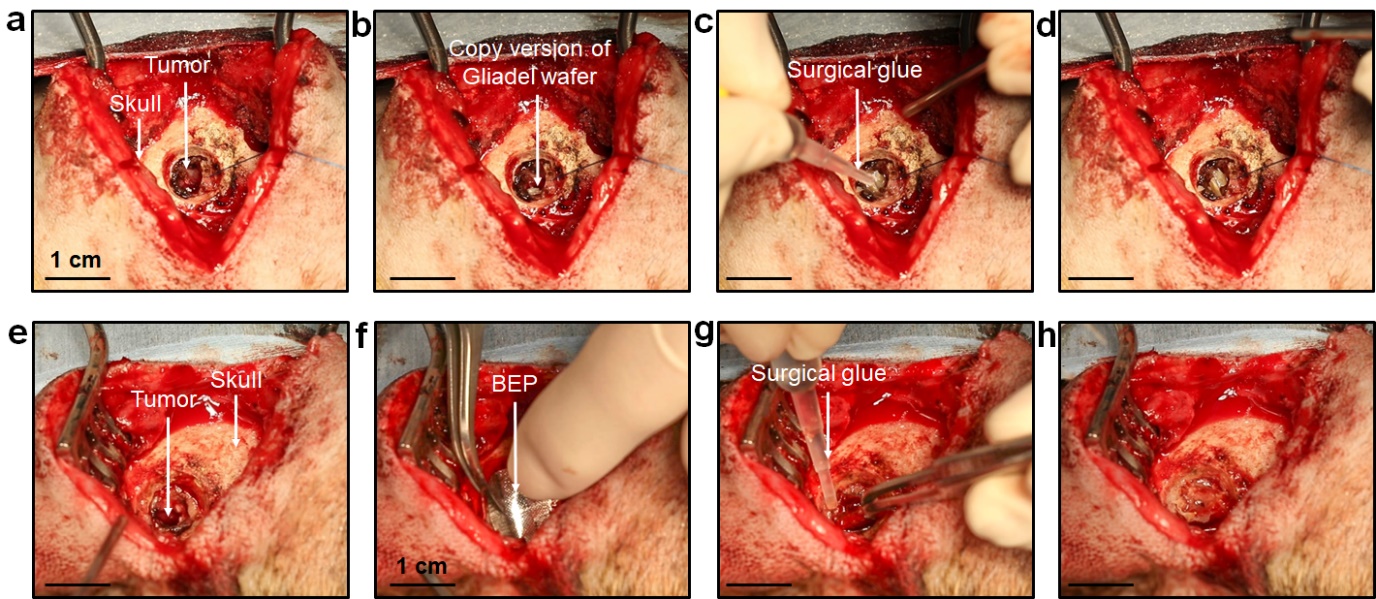


**Supplementary Figure 24**

Surgical process for tumor resection and BEP implantation in the canine brain model.

Optical camera images show (**a**) before and (**b**) during implantation of the control wafer; (**c**) before and (**d**) after surgical glue injection; (**e**) before and (**f**) during BEP implantation; and (**g**) before and (**h**) after surgical glue injection.


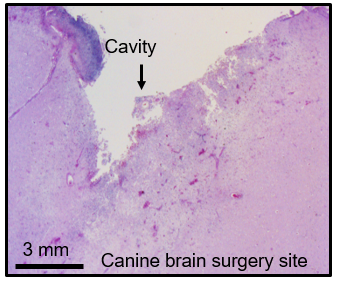


**Supplementary Figure 25**

Histology images of tissues (H&E) near the surgery site, which show the residual brain tumor infiltrated in the normal brain tissue.


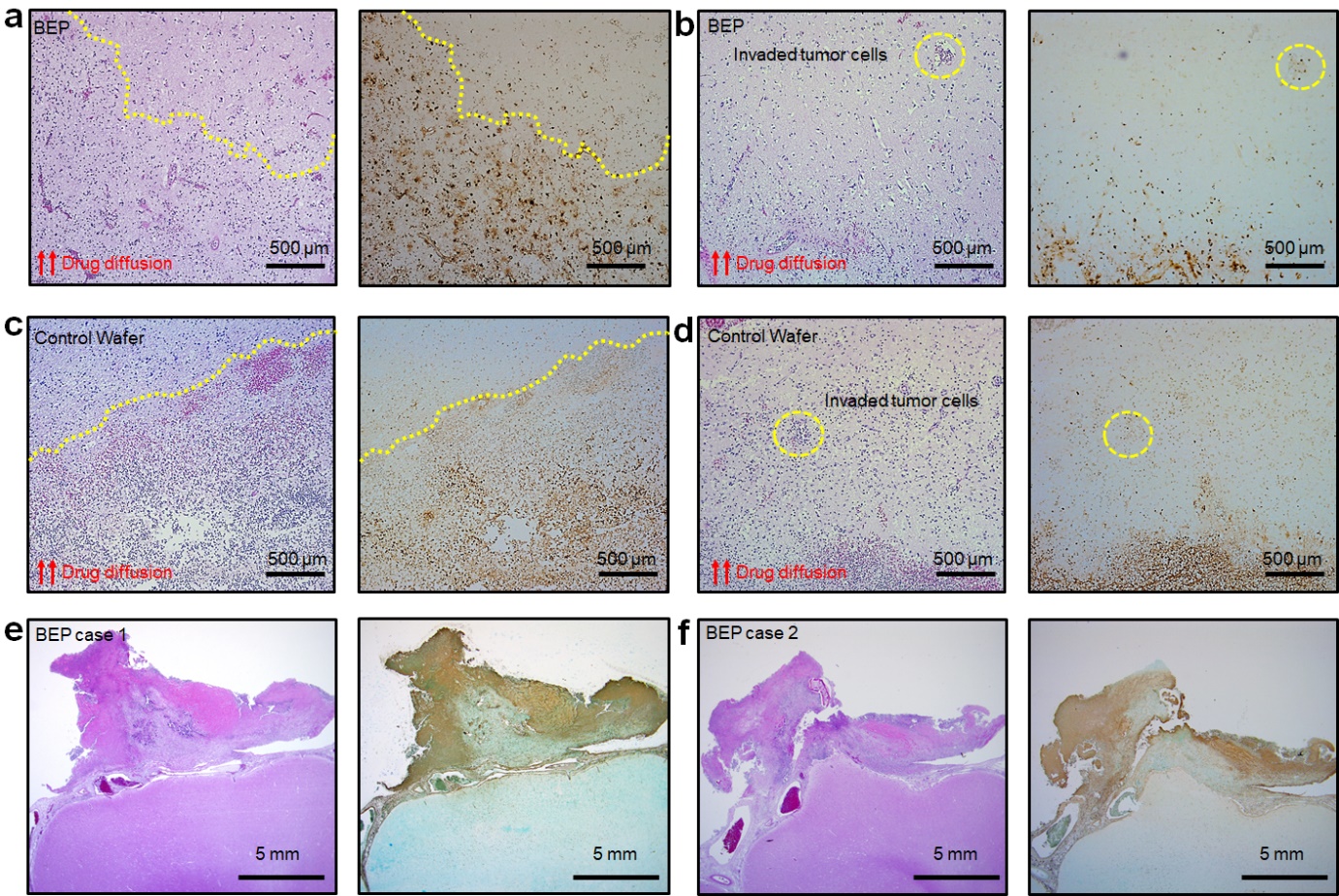


**Supplementary Figure 26**

Tumor specific death confirmed by H&E stain and TUNEL assay on same site in canine brain tumor model. (**a**) H&E stain (left) and TUNEL assay (right) of cavity which is treated by BEP after 2 days. (**b**) The dead invaded tumor cells with the BEP treatment. (**c**) H&E stain (left) and TUNEL assay (right) of tissues at the cavity after 2 days, which is treated by the control wafer. (**d**) The image of the untreated tumor cells in the control wafer case. (**e**), (**f**) H&E stained (left) and TUNEL assay (right) of the other site of the tumor cavity, which are treated by BEP after 1 week.

**Supplementary Tables**

|  | IV | Heating | OST | Control wafer |
| --- | --- | --- | --- | --- |
| IV | - | - | - | - |
| Heating | NS | - | - | - |
| OST | *p* = 0.0172 | NS | - | - |
| Control wafer | *p* = 0.0025 | NS | NS | - |
| OST+Heating | *p* = 0.0024 | *p* = 0.0024 | *p* = 0.019 | *p* = 0.0048 |

**Supplementary Table 1**

Statistical significance of the differences between the final tumor volumes in the indicated experimental groups, measured by 9.4 T magnetic resonance imaging. (IV, mice received an intravascular injection of DOX; Heating, mice received BEP and mild-thermic actuation, without DOX; OST, mice received BEP containing DOX; Control wafer, mice received Control wafer containing carmustine; OST+Heating, mice received BEP with DOX and mild-thermic actuation; NS, not significant, *p* > 0.05)

The comparison of final volume of all concerned mice in each group were evaluated by Mann-Whitney U test. The *p* value for individual tests was multiplied by the number of comparisons made (Bonferroni correction).

|  | IV | Heating | OST | Control wafer |
| --- | --- | --- | --- | --- |
| IV | - | - | - | - |
| Heating | NS | - | - | - |
| OST | NS | *p* = 0.0056 | - | - |
| Control wafer | *p* = 0.0196 | *p* = 0.0044 | NS | - |
| OST+Heating | *p* = 0.0006 | *p* = 0.0008 | *p* = 0.036 | *p* = 0.013 |

**Supplementary Table 2**

Statistical significance of the differences between survival rates in the indicated experimental groups. IV, mice received an intravascular injection of doxorubicin (DOX); Heating, mice received bioresorbable electronic patch (BEP) and mild-thermic actuation, without DOX; OST, mice received BEP containing DOX; Control wafer, mice received Control wafer containing carmustine; OST+Heating, mice received BEP with DOX and mild-thermic actuation; NS, not significant, *p* value > 0.05)

The log-rank test was used to compare survival plot. The *p* value for individual tests was multiplied by the number of comparisons made (Bonferroni correction).

| Elements | Thickness (μm) | Heat capacity  (J kg^-1^ K^-1^) | Heat conductivity  (W m^-1^ K^-1^) | Mass density  (kg m^-3^) | Products |
| --- | --- | --- | --- | --- | --- |
| Cerebrospinal fluid | 2000 | 4187 | 0.62 | 1000 |  |
| Top PLGA | 5~100 | 1800 | 0.13 | 1300 | Lactic acid |
|  |  |  |  |  | Glycolic acid |
| Magnesium | 3 | 1020 | 156 | 1738 | Mg^2+^ |
| Bottom PLA | 7 | 1800 | 0.13 | 1300 | Lactic acid |
| Starch (77 % *w/w*) |  | 1160 | 0.38 | 1500 | Glucose |
| Glycerol (23 % *w/w*) |  | 2413 | 0.29 | 1260 | Glycerol |
| Drug reservoir | 200 | 1449 | 0.36 | 1445 |  |
| Water (77 % *w/w*) |  | 4187 | 0.62 | 1000 |  |
| Protein (9 % *w/w*) |  | 1500 | 0.2 | 1450 |  |
| Fat (16% *w/w*) |  | 2674 | 0.19 | 1000 |  |
| Brain tissue (White matter) | Infinite | 3700 | 0.51 | 1030 |  |
| Doxorubicin | (1mg) |  |  |  | Doxorubicin |

**Supplementary Table 3**

Thermophysical properties, thicknesses, and biodegradation products of the BEP layers.

|  | Symbol | Unit | Value |
| --- | --- | --- | --- |
| Relative permeability | $\mu_{r}$ | 1 | 1 |
| Relative permittivity | $\epsilon_{r}$ | 1 | 850 |
| Electric conductivity | $\sigma$ | S m^-1^ | 0.03 |
| Heat capacity (constant pressure) | $C_{p}$ | J kg^-1^ K^-1^ | 1000 |
| Density | $\rho$ | Kg m^-3^ | 1.9 |
| Thermal conductivity | $k$ | W m^-1^ K^-1^ | 0.3 |

**Supplementary Table 4**

Electromagnetic properties of skull used in 3D FEM simulations.

**Supplementary References**

1. Mills, J. K. & Needham, D. Temperature triggered nanotechnology for chemotherapy: Rapid release from lysolipid temperature-sensitive liposomes. *NSTI-Nanotech* **2**, 5-8 (2006).

2. Gabriel, S., Lau, R. W. & Gabriel, C. The dielectric properties of biological tissues: III. Parametric models for the dielectric spectrum of tissues. *Phys. Med. Biol.* **41**, 2271-2293 (1996).

3. El-Brawany, M. A. *et al.* Measurement of thermal and ultrasonic properties of some biological tissues. *J. Med. Eng. Technol*. **33**, 249-256 (2009).

4. White, D. N., Curry, G. R. & Stevenson, R. J. The acoustic characteristics of the skull. *Ultrasound Med. Biol*. **4**, 225-252 (1978).

5. Krisht, A. F., Yoo, K., Arnautovic, K. I. & Al-Mefty, O. Cavernous sinus tumor model in the canine: A simulation model for cavernous sinus tumor surgery. *Neurosurgery* **56**, 1361-1366 (2005).

6. Whelan, H. T., Clanton, J. A., Wilson, R. E., Tulipan, N. B. Comparison of CT and MRI brain tumor imaging using a canine glioma model. *Pediatr. Neurol.* **4**, 279-283 (1988).

7. Johnston, M. The importance of lymphatics in cerebrospinal fluid transport. *Lymph. Res. Biol.* **1**, 41-45 (2003).

8. Jeon, D. *et al.* A cell-free extract from human adipose stem cells protects mice against epilepsy. *Epilepsia* **52**, 1617-1626 (2011).

9. Lee, S.-T. *et al.* Slowed progression in models of huntington disease by adipose stem cell transplantation. *Ann. Neurol.* **66**, 671-681 (2009).

10. Lee, S.-T. *et al.* Inhibition of miR-203 reduces spontaneous recurrent seizures in mice. *Mol. Neurobiol.* **54**, 3300-3308 (2017).
