# Supplementary material for: Flexible, sticky, and biodegradable wireless device for drug delivery to brain tumors
Source: Nat Commun. 2019 Nov 15;10:5205. doi: 10.1038/s41467-019-13198-y (PMC6858362; doi:10.1038/s41467-019-13198-y)
Supplement: Supplementary file 3 — Reporting Summary [file 41467_2019_13198_MOESM3_ESM.pdf]

nature research

Corresponding author(s): Dae-Hyeong Kim

Last updated by author(s): Aug 28, 2019

Reporting Summary

Nature Research wishes to improve the reproducibility of the work that we publish. This form provides structure for consistency and transparency in reporting. For further information on Nature Research policies, see Authors & Referees and the Editorial Policy Checklist .

Statistics

For all statistical analyses, confirm that the following items are present in the figure legend, table legend, main text, or Methods section.

n/a	Confirmed	
☐	☒	The exact sample size (n) for each experimental group/condition, given as a discrete number and unit of measurement
☐	☒	A statement on whether measurements were taken from distinct samples or whether the same sample was measured repeatedly
☐	☒	The statistical test(s) used AND whether they are one- or two-sided<br>*Only common tests should be described solely by name; describe more complex techniques in the Methods section.*
☒	☐	A description of all covariates tested
☐	☒	A description of any assumptions or corrections, such as tests of normality and adjustment for multiple comparisons
☒	☐	A full description of the statistical parameters including central tendency (e.g. means) or other basic estimates (e.g. regression coefficient) AND variation (e.g. standard deviation) or associated estimates of uncertainty (e.g. confidence intervals)
☐	☒	For null hypothesis testing, the test statistic (e.g. F, t, r) with confidence intervals, effect sizes, degrees of freedom and P value noted<br>*Give P values as exact values whenever suitable.*
☒	☐	For Bayesian analysis, information on the choice of priors and Markov chain Monte Carlo settings
☒	☐	For hierarchical and complex designs, identification of the appropriate level for tests and full reporting of outcomes
☒	☐	Estimates of effect sizes (e.g. Cohen's d, Pearson's r), indicating how they were calculated

Our web collection on statistics for biologists contains articles on many of the points above.

Software and code

Policy information about availability of computer code

Data collection	Not applicable.
Data analysis	Not applicable.

For manuscripts utilizing custom algorithms or software that are central to the research but not yet described in published literature, software must be made available to editors/reviewers. We strongly encourage code deposition in a community repository (e.g. GitHub). See the Nature Research guidelines for submitting code & software for further information.

Data

Policy information about availability of data

All manuscripts must include a data availability statement. This statement should provide the following information, where applicable:

- Accession codes, unique identifiers, or web links for publicly available datasets
- A list of figures that have associated raw data
- A description of any restrictions on data availability

The data that support the findings of this study are available from the corresponding author upon reasonable request.

Field-specific reporting

Please select the one below that is the best fit for your research. If you are not sure, read the appropriate sections before making your selection.

☒ Life sciences ☐ Behavioural & social sciences ☐ Ecological, evolutionary & environmental sciences

For a reference copy of the document with all sections, see nature.com/documents/nr-reporting-summary-flat.pdf

Life sciences study design

All studies must disclose on these points even when the disclosure is negative.

Sample size	All sample size is described in text.
Data exclusions	No data was excluded from analyses.
Replication	All measures are done replicately.
Randomization	All of samples are selected randomly in whole experiments.
Blinding	Not applicable.

Reporting for specific materials, systems and methods

We require information from authors about some types of materials, experimental systems and methods used in many studies. Here, indicate whether each material, system or method listed is relevant to your study. If you are not sure if a list item applies to your research, read the appropriate section before selecting a response.

Materials & experimental systems

n/a	Involved in the study	
☒	☐	Antibodies
☒	☐	Eukaryotic cell lines
☒	☐	Palaeontology
☐	☒	Animals and other organisms
☒	☐	Human research participants
☒	☐	Clinical data

Methods

n/a	Involved in the study	
☒	☐	ChIP-seq
☐	☒	Flow cytometry
☐	☒	MRI-based neuroimaging

Animals and other organisms

Policy information about studies involving animals; ARRIVE guidelines recommended for reporting animal research

Laboratory animals	BALB/C nude, normal mice and mongrel dogs were involved for experiment.
Wild animals	Not applicable.
Field-collected samples	Not applicable.
Ethics oversight	This study was approved by our Institutional Animal Care and Use Committee (IACUC; No. 14-0156-C1A3) and was performed in accordance with our IACUC guidelines and with the National Institute of Health Guide for the Care and Use of Laboratory Animals.

Note that full information on the approval of the study protocol must also be provided in the manuscript.

Flow Cytometry

Plots

Confirm that:

☒ The axis labels state the marker and fluorochrome used (e.g. CD4-FITC).

☒ The axis scales are clearly visible. Include numbers along axes only for bottom left plot of group (a 'group' is an analysis of identical markers).

☒ All plots are contour plots with outliers or pseudocolor plots.

☒ A numerical value for number of cells or percentage (with statistics) is provided.

Methodology

Sample preparation	We used cells treated with DOX and analyzed wavelength of DOX using FACS.
Instrument	BD LSRII (SORP)
Software	We used software which is CellQuest Pro.

Cell population abundance | For this study, we used a cell line and checked homogeneity including cell size and condition before starting FACS analysis.

Gating strategy | We used same number of cells and confirmed the number once again using FACS. The boundary between control and experimental was determined using positive and negative control.

☒ Tick this box to confirm that a figure exemplifying the gating strategy is provided in the Supplementary Information.

Magnetic resonance imaging

Experimental design

Design type	Not applicable.
Design specifications	Not applicable.
Behavioral performance measures	Not applicable.

Acquisition

Imaging type(s)	structural
Field strength	3T and 9.4T
Sequence & imaging parameters	For 3T imaging, the brain imaging sequences included axial turbo-spin echo T2-weighted images (T2WI), axial gradient-echo T1-weighted images (T1WI), and axial contrast-enhanced (CE) T1WI. After routine localization images were obtained, T2WI (repetition time/echo time, 5160 ms/91 ms; flip angle 131°, section thickness, 5 mm; matrix, 640×290) and T1WI (repetition time/echo time, 990 ms/9.8 ms; flip angle 70°, section thickness, 1.5 mm; matrix, 384×212) were acquired. Subsequently, CE T1WI was acquired after an intravenous injection of 0.2 mL/kg godoteric acid (Dotarem; Guerbet, France) via the cephalic vein.<br>For 9.4T imaging, a millipede 1-ch coil was used for both radio frequency transmission and signal reception (Agilent Technologies, USA) and a fast spin echo sequence was used to produce a T2-weighted image. The measurement parameters were as follows: repetition time = 3000 ms; effective echo time = 30.82 ms; field-of-view = 20×35 mm; echo train length = 4; matrix = 256×256; slice thickness = 1.0 mm.
Area of acquisition	Whole brain imaging was acquired for a dog model by using 3T MRI scanner, and only tumor area was scanned for a mouse model by using 9.4T MRI.

Diffusion MRI ☐ Used ☒ Not used

Preprocessing

Preprocessing software	Not applicable.
Normalization	Not applicable.
Normalization template	Not applicable.
Noise and artifact removal	Not applicable.
Volume censoring	Not applicable.

Statistical modeling & inference

Model type and settings	Not applicable.
Effect(s) tested	Not applicable.

Specify type of analysis: ☐ Whole brain ☐ ROI-based ☐ Both

Statistic type for inference<br>(See Eklund et al. 2016)	Not applicable.
Correction	Not applicable.

Models & analysis

n/a	Involved in the study
☒	☐ Functional and/or effective connectivity
☒	☐ Graph analysis
☒	☐ Multivariate modeling or predictive analysis
